# Evolutionary History of *Rhus chinensis* (Anacardiaceae) From the Temperate and Subtropical Zones of China Based on cpDNA and Nuclear DNA Sequences and Ecological Niche Model

**DOI:** 10.3389/fgene.2019.00171

**Published:** 2019-03-05

**Authors:** Yukang Liang, Yang Zhang, Jun Wen, Xu Su, Zhumei Ren

**Affiliations:** ^1^School of Life Science, Shanxi University, Taiyuan, China; ^2^Natural History Research Center, Shanghai Natural History Museum, Branch of Shanghai Science and Technology Museum, Shanghai, China; ^3^Department of Botany, National Museum of Natural History, Smithsonian Institution, Washington, DC, United States; ^4^Key Laboratory of Medicinal Animal and Plant Resources of the Qinghai-Tibetan Plateau in Qinghai Province, School of Life Science, Qinghai Normal University, Xining, China

**Keywords:** *Rhus chinensis*, evolutionary history, DNA sequences, China’s temperate and subtropical zone, ecological niche modeling

## Abstract

To explore the origin and evolution of local flora and vegetation, we examined the evolutionary history of *Rhus chinensis*, which is widely distributed in China’s temperate and subtropical zones, by sequencing three maternally inherited chloroplast DNAs (cpDNA: *trn*L*-trn*F, *psb*A-*trn*H, and *rbc*L) and the biparentally inherited nuclear DNA (nuDNA: *LEAFY*) from 19 natural populations of *R. chinensis* as well as the ecological niche modeling. In all, 23 chloroplast haplotypes (M1–M23) and 15 nuclear alleles (N1–N15) were detected. The estimation of divergence time showed that the most recent common ancestor dated at 4.2 ± 2.5 million years ago (Mya) from cpDNA, and the initial divergence of genotypes occurred at 4.8 ± 3.6 Mya for the nuDNA. Meanwhile, the multimodality mismatch distribution curves and positive Tajima’s *D* values indicated that *R. chinensis* did not experience population expansion after the last glacial maximum. Besides, our study was also consistent with the hypothesis that most refugia in the temperate and subtropical zones of China were *in situ* during the glaciation.

## Introduction

The Quaternary climate oscillations occurred in the past ca. 2.58 million years ago (Mya) have resulted in several glacial and interglacial cycles in the Northern Hemisphere (Shackleton and Opdyke, 1973). These climatic alterations have left imprints in geographical distributions, population structures, and demographic histories of plant and animal species (Abbott et al., 2000; Avise, 2000; Hewitt, 2004, 2011; Qiu et al., 2011, 2013; Wen et al., 2014, 2016), which can be traced by analyses of genetic variations within and between extant populations (Abbott et al., 2000; Johansen and Latta, 2003; Hewitt, 2004). In Europe and North America, the fossil records of plant species and phylogeographic analyses had indicated common patterns of geographical range shifts that plants retreated southward and to lower elevations during glacial periods and while recolonized rapidly the northern areas and higher elevations during the interglacial and postglacial periods (Nason et al., 2002; Petit et al., 2003; Stewart et al., 2010; Sakaguchi et al., 2011; Segovia et al., 2012; Voss et al., 2012; Tzedakis et al., 2013; de Lafontaine et al., 2014). While in China, especially the Qinghai-Tibet Plateau (QTP) and adjacent regions, considerable research achievements have also been attained on inferring the Quaternary phylogeographic histories of plant species based on the approach of population genetics (e.g., Zhang et al., 2005, 2015; Meng et al., 2007; Chen K.M. et al., 2008; Yang et al., 2008; Wang et al., 2009; Opgenoorth et al., 2010; Xu et al., 2010; Qiu et al., 2011; Zou et al., 2012; Wang G.N. et al., 2014; Wen et al., 2014; Liu Y.P. et al., 2015; Wan et al., 2016).

The temperate and subtropical region of China is a model area for studying plant species in response to past climate changes (Chen S.C. et al., 2012; Li X.H. et al., 2012; Qi et al., 2012; Zhao et al., 2013; Fu et al., 2014). Up to date, many phylogeographic studies have been used to elucidate the impacts of the uplifts of the QTP on the climate within the modern-day temperate and subtropical zones, or warm temperate zones in China (e.g., Yellow River Basin, Chen K.M. et al., 2008 and Chen Y.Y. et al., 2008; Yunnan-Guizhou Plateau, Fan et al., 2013; Wang W. et al., 2014; Yangtze River, Sun et al., 2013; Wang H. et al., 2015; Qinling Mountains, Liu J.Q. et al., 2014; Lu et al., 2016; QTP, Liu D. et al., 2015 and Liu Y.P. et al., 2015); i.e., 23.5°–42.0° N and 98.0°–124.0° E (Gao et al., 2003; Shangguan et al., 2009). The results showed that the QTP acted as a barrier against glaciation within the warm temperate zones of China and resulted in the arid climate for thousands of years within the Quaternary period, which has been widely accepted nowadays (Wang et al., 2013; Yu et al., 2013; Meng et al., 2014). Thus, the present warm temperate region probably served as a glacial refugia for plant species in the past time, and this hypothesis has been tested and advanced through phylogeographic studies (e.g., Li Y. et al., 2012; Liu et al., 2012; Qi et al., 2012; Wan et al., 2016). However, it is less well known whether population genetic diversification of plants within the warm temperate zone or within the glacial refugia is due to isolation on a heterogeneous landscape or adaptation and selection along ecological gradients (Su et al., 2015; Zhao et al., 2016). Therefore, more phylogeographic studies of additional plant species within the warm temperate refugial regions are necessary in order to detect their spatial geographic patterns and to assess the underlying causes.

*Rhus chinensis* belongs to the plant family Anacardiaceae and is a common deciduous tree that is endemic to the warm temperate zone of Asia. It widely occurs at the elevation of 170–2700 m above sea level in Shaanxi, Shanxi, Hebei, Sichuan, Hunnan, and Yunnan of China (Zheng and Min, 1980). Due to its commonality and widespread distribution within the warm temperate zone, *R. chinensis* is thus an ideal study case for phytogeography within this region. In this study, we used three cpDNA regions (*trn*L*-trn*F, *psb*A-*trn*H, and *rbc*L) and one nuDNA region (*LEAFY*) to examine (1) the genetic diversity and structure of *R. chinensis* populations in China and (2) how is the demographic history of *R. chinensis* during the Quaternary climate oscillations, and further to explore the origin and evolution of local flora and vegetation.

## Materials and Methods

### Population Sampling

**FIGURE 1:**
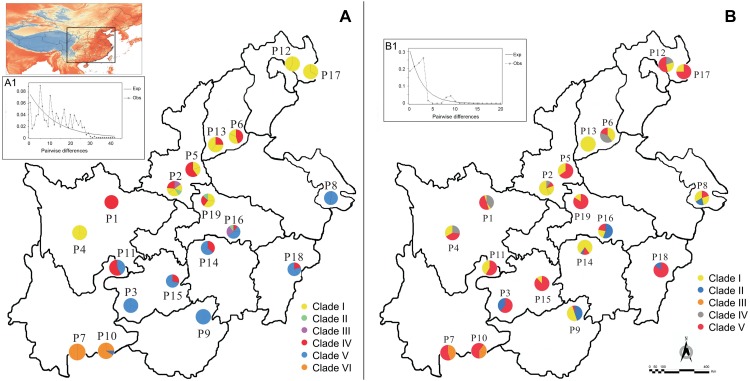
Geographic distribution of haplotypes and genotypes for *Rhus chinensis* based on cpDNA **(A)** and nuDNA **(B)**. Multimodality mismatch distribution curves of cpDNA and nuDNA in the overall populations are shown in **A1** and **B1**.

In total, leaf samples of 312 individuals were collected from 19 natural populations of *R. chinensis*, representing its whole geographic distribution within the warm temperate zone of China (see Figure 1 and Table 1). Eight to 20 individuals were collected for each population, and all individuals were at least 15 m apart. We obtained several voucher specimens for each population, which were deposited at the School of Life Sciences, Shanxi University, Taiyuan, Shanxi, China. The information of latitude, longitude, and altitude of each population were recorded using an Etrex GIS (Garmin, Taiwan, China).

**Table 1:** Sampling of *Rhus chinensis* in the present study.

Population	Locality	No. of samples	Date	Longitude(E)	Latitude(N)	Altitude (m)
P1	Anxian, SC	20	2009.9	104°54′	31°57′	1009
P2	Chenggu, SAX	20	2013.8	107°59′	33°09′	1453
P3	Danzhai, GZ	17	2007.7	107°47′	26°11′	1005
P4	Emei, SC	15	2009.9	103°31′	29°51′	1100
P5	Huxian, SAX	20	2013.8	108°50′	33°32′	605
P6	Jincheng, SX	11	2009.8	112°44′	35°38′	800
P7	Jinping, YN	8	2008.10	103°13′	23°46′	1248
P8	Tianpingshan, JS	19	2009.9	120°50′	31°28′	200
P9	Longsheng, GX	20	2008.9	110°01′	25°47′	702
P10	Malipo, YN	10	2008.10	104°42′	23°07′	1619
P11	Shuifu, YN	12	2007.7	110°14′	28°37′	1644
P12	Sanhe, HEB	16	2009.8	117°00′	39°58′	877
P13	Sijiao, SX	20	2010.8	111°40′	35°08′	1100
P14	Sangzhi, HN	20	2010.8	110°17′	29°40′	800
P15	Taijiang, GZ	17	2007.7	108°19″	26°39′	721
P16	Wufeng, HUB	20	2012.9	110°40′	30°12′	614
P17	Xinglong, HEB	20	2009.8	117°58′	40°38′	960
P18	Yanshan, JX	12	2013.8	117°80′	27°50′	1212
P19	Zhushan, HUB	15	2013.8	110°14′	32°13′	717

*Notes: the capital abbreviations represent the different provinces, SC, Sichuan; SAX, Shaanxi; GZ, Guizhou; SX, Shanxi; YN, Yunnan; JS, Jiangsu; GX, Guangxi; HEB, Hebei; HN, Hunan; HUB, Hubei; JX, Jiangxi.*

The species from Anacardiaceae were used as outgroups. The cpDNA sequences of four species were downloaded from GenBank, *Rhus virens* (EF682861, KF664327, KF664558), *Rhus typhina* (AY640446, HQ427036, HQ590236), *Rhus glabra* (AY640440, KF664325, KX397919), and *Pistacia vera* (EF193139, KF664307, AJ235786). There are no nuclear sequences for the above four species in GenBank, so we used another two species *Mangifera indica* (GU338039) and *P. chinensis* (KC174710) as the outgroups in the nuDNA analysis.

### DNA Sequencing

Total genomic DNAs from approximately 20 mg of silica gel-dried leaf materials were extracted using a Plant Genomic DNA kit (Tiangen Biotech, Beijing, China), and three cpDNA fragments (*trn*L*-trn*F, *psb*A-*trn*H, and *rbc*L) and one nuclear gene (*LEAFY*) were amplified and sequenced by the following primers: *trn*L*-trn*F (5′-CGAAATCGGTAGAGGCTACG-3′; 5′-ATTTGAACTGGTGACACGAG-3′; Taberlet et al., 1991), *psb*A*-trn*H (5′-GTTATGCATFAACGTAATGCTC-3′; 5′-CGCGCATGGTGGATTCACAAATC-3′; Sang et al., 1997), *rbc*L (5′-ATGTCACCACAAACAGAGAC-3′; 5′-TCAAATTCAAACTTGATTTCTTTC-3′; Little and Barrington, 2003), *LEAFY* (5′-TACACGGCAGCGAAGATAGC-3′; 5′-CTAGAAGCAGCGGCACTATTG-3′; Oh and Potter, 2003). Polymerase chain reaction (PCR) was performed in a volume of 50 μL and each reaction contained 30–50 ng genomic DNA, 25 μL amplification reaction mixture (PCR mix kit, Tiangen Biotech, Beijing, China), and 20 μmol/L primers, and under the following conditions: initial denaturation at 94°C for 3 min, 35 cycles of 30 s at 94°C, 30 s at 54–60°C, 90 s at 72°C, and a final extension step of 7 min at 72°C. All the qualified PCR products were sent to Majorbio Bio-pharm Technology Co., Ltd. (Shanghai) for sequencing.

### Data Analysis

We aligned sequences with Clustal_X (Thompson et al., 1997) and coded indels following the method of Simmons and Ochoterena (2000). Indels within mononucleotide repeat regions were deleted for phylogenetic analyses, because the homology of these indels could not be verified (Chen S.C. et al., 2012).

The levels of inter- and intra-population genetic diversity (*h*: haplotype diversity and *π:* nucleotide diversity) were calculated for the cpDNA and nuDNA using DnaSP version 5.0 (Rozas et al., 2003). We compared *G*_ST_ and *N*_ST_ using the U-statistic, which is approximated by a Gaussian variable by taking into account the covariance between *G*_ST_ and *N*_ST_, and a one-sided test (Pons and Petit, 1996). The former considers only haplotype frequencies while *N*_ST_ also takes into account differences between haplotypes. When *N*_ST_ is larger than *G*_ST_, phylogeographic structure is obvious, which indicates that closely related haplotypes were found more often in the same area than less closely related haplotypes (Pons and Petit, 1996). We also estimated genetic differentiation among all populations with AMOVA and inferred population growth and expansion according to Tajima’s *D* using Arlequin version 3.0 (Excoffier et al., 2005), with 1000 random permutations to test for significance of partitions. Genealogical relationships among cpDNA and nuDNA haplotypes were constructed using TCS version 1.21 (Clement et al., 2000).

The phylogenetic relationships among haplotypes and genotypes of cpDNA and nuDNA were reconstructed with Bayesian inference (BI) methods in MrBayes version 3.1.2 (Ronquist and Huelsenbeck, 2003). We applied the best fit model, GTR + I + G, which was inferred by Modeltest 3.7 under the Akaike information criterion (Posada and Crandall, 1998; Ronquist and Huelsenbeck, 2003). The BI consisted of two parallel runs with four incrementally heated chains and three million generations sampled every 1000 generation. The output was diagnosed for convergence using TRACER v.1.3 (Rambaut and Drummond, 2007), and summary statistics and trees were generated using the last two million generation in MrBayes version 3.1.2 (Ronquist and Huelsenbeck, 2003). In order to distinguish the haplotypes and genotypes clearly, the branches with high bootstrap value (>0.95) were classified as new clades based on the phylogenetic trees (Porter et al., 2005; Pyron, 2011; Ye et al., 2017).

The divergence times within *R. chinensis* were estimated using a molecular clock and fossil data. Three fossils of *Rhus* were used to calibrate the node ages of *R. typhina* and *R. glabra* (6.0 Mya), *R. typhina* and *R. virens* (38.1 Mya), and *R. typhina* and *P. vera* (49.1 Mya) for cpDNA data, respectively (Yi et al., 2004), while one fossil (49.1 Mya) was used as the divergence time between *Rhus* and *Pistacia* for nuDNA data. Both the strict and relaxed molecular clock rates were tested in MEGA6 (Tamura et al., 2013) using the BI summary tree, and they could not be rejected for either the cpDNA or nuDNA data. Therefore, the strict and relaxed clocks were both applied to the two datasets in the BASEML and MCMCTREE programs of PAML (Yang, 2007), and used our BI summary tree as the guide tree.

### Ecological Niche Modeling

We compared the current distributions of *R. chinensis* with its inferred distributions during the last glacial maximum (LGM; ∼21,000 years BP) with ecological niche modeling in Maxent version 3.3.3 (Phillips et al., 2006). To perform this modeling, we first obtained the geocoordinates of 73 occurrence data of *R. chinensis* from the Chinese Virtual Herbarium^1^ and Global Biodiversity Information Facility^2^. Subsequently, we constructed the models using 19 bioclimatic variables from the WorldClim database^3^ (Hijmans et al., 2005) representing the present (averaged from 1950) and the LGM according to the Community Climate System Model (CCSM; Collins et al., 2006). We employed 20 replicates based on 80% of the distribution coordinates for training and 20% for testing, and adopted the model with the best AUC values (Phillips et al., 2006). We performed a jackknife test to estimate the percent contributions of bioclimatic variables to the prediction for the distributional models. Meanwhile, we also employed the “10 percentile presence” threshold logistic approach as determined by Maxent in order to distinguish the threshold between suitable and unsuitable habitats for further analyses. We drew Graphics for each predicted SDM using DIVA-GIS 7.5 (Hijmans et al., 2005).

## Results

### Genetic Diversity and Structure

Aligned cpDNA dataset consisted of 2051 bp with 70 nucleotide substitutions and two indels. We detected 23 different haplotypes (M1–M23) based on combined cpDNA dataset from 19 populations. The *LEAFY* gene region varied from 412 to 645 bp and had an aligned length of 682 bp, which contained 14 nucleotide substitutions. Our sequences of *LEAFY* comprised 15 genotypes (N1–N15). Based on cpDNA and nuDNA sequences, the total haplotype diversity of *R. chinensis* was estimated to be 0.738 and 0.614, and the total nucleotide diversity was inferred to be 6.910 × 10^−3^ and 3.050 × 10^−3^, respectively (Table 2). We found the highest levels of haplotype and nucleotide diversity in four populations: P2, P11, P14, and P16 (Table 2). The most widespread haplotypes and genotypes were M1 (in 11 of 19 populations, cpDNA) and N3 (in 18 of 19 populations, nuDNA; Table 2), respectively. Based on cpDNA and nuDNA sequences, M1 and N3 were the primary haplotype and genotype, respectively (Figure 1).

**Table 2:** The total haplotype and nucleotide diversity of *Rhus chinensis*.

	cpDNA			nuDNA		
Population	*h*	π × 10^−3^	Haplotypes (No.)	*H*	π × 10^−3^	Genotypes (No.)
P1	0.000	0.000	M1(20)	0.616	1.140	N1(8), N2(1), N3(10), N4(1)
P2	0.719	8.810	M1(5), M2(10), M3(2), M4(3), M8(4), M21(2)	0.686	3.870	N1(2), N3(2), N5(4), N7(1), N15(11)
P3	0.550	5.230	M5(7), M6(4), M7(2)	0.422	2.270	N3(10), N14(7)
P4	0.586	5.370	M2(4), M3(4), M4(7)	0.505	2.570	N1(4), N3(6), N4(5)
P5	0.358	5.620	M1(16), M2(2), M9(2)	0.379	1.410	N3(13), N15(7)
P6	0.591	5.400	M1(5), M10(4), M11(2)	0.485	2.140	N1(4), N3(2), N7(5)
P7	0.571	3.520	M12(4), M13(4)	0.314	2.120	N3(4), N10(2)
P8	0.526	2.590	M14(10), M15(9)	0.653	2.180	N3(4), N6(3), N11(6), N12(6)
P9	0.589	5.700	M5(4), M16(4), M17(12)	0.605	2.580	N3(1), N4(7), N6(1), N14(5)
P10	0.400	4.970	M12(3), M13(6), M18(1)	0.389	2.980	N3(6), N9(2), N10(2)
P11	0.636	6.560	M1(1), M5(1), M6(3), M7(7)	0.236	1.360	N3(7), N4(5)
P12	0.533	2.730	M19(9), M20(7)	0.321	2.540	N1(3), N4(4), N7(9)
P13	0.689	6.320	M1(5), M2(7), M19(8)	0.000	0.000	N13(20)
P14	0.554	5.690	M1(8), M5(5), M6(6), M23(5)	0.300	2.150	N3(3), N4(19), N6(1)
P15	0.694	8.220	M1(5), M6(1), M17(6), M18(2)	0.221	0.970	N3(15), N4(2)
P16	0.645	8.700	M1(2), M6(3), M8(5), M21(3), M23(8)	0.774	3.000	N3(4), N4(9), N6(3), N14(11)
P17	0.442	2.260	M19(6), M20(14)	0.395	1.740	N3(15), N4(5)
P18	0.318	2.510	M1(2), M22(2), M23(5)	0.268	1.400	N3(10), N14(2)
P19	0.681	7.820	M1(4), M2(5), M4(4), M21(2)	0.281	1.830	N3(12), N7(3)
Total	0.738	6.910		0.614	3.050	

*π: nucleotide diversity, h: haplotype diversity.*

AMOVA analysis indicated that genetic variation in *R. chinensis* was greater within populations than among them (*P* < 0.01; Table 3). The mismatch distribution (Figure 1A1,B1) and positive values of Tajima’s *D* value (1.19, 0.05 < *P* < 0.10 for cpDNA; 2.37, *P* < 0.01 for nuDNA) of all populations rejected a sudden expansion model, and positive Tajima’s *D* may indicate population admixture. Phylogeographic structure is not obvious at the species level for both sets of genetic markers. For the cpDNA data, *N*_ST_ (0.382) was slightly higher than *G*_ST_ (0.375), while for the *LEAFY*, the difference between the two indices was not significant (*N*_ST_ = 0.321, *G*_ST_ = 0.319, *P* > 0.05).

**Table 3:** Analysis of molecular variance (AMOVA) of *Rhus chinensis* populations based on nucleotide sequences.

Gene types	Source of variation	*d.f.*	*SS*	*VC*	*PV*	*F*_ST_
**Chlorotype**						0.63931^∗^
	Among regions	4	1425.914	4.95009	30.05	
	Among populations	14	1396.234	5.58051	33.88	
	Within populations	293	1740.808	5.94132	36.07	
	Total	311	4562.955	16.47192		
**Genotypes**						0.70675^∗^
	Among regions	3	321.790	1.02156	29.33	
	Among populations	15	146.323	0.5.815	14.59	
	Within populations	293	427.270	1.95386	56.09	
	Total	311	895.383	3.48357		

*Notes: d.f., degrees of freedom; SS, sum of squares; VC, variance components; F_ST_, correlation within populations relative to total; ^∗^P < 0.01.*

### Phylogeography and Divergence Time

**FIGURE 2:**
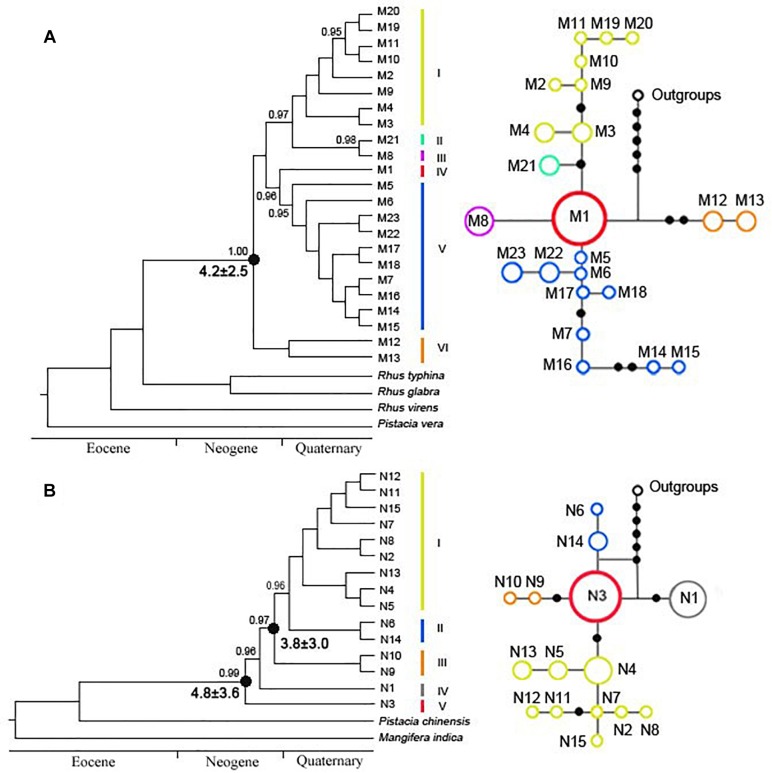
Phylogenetic trees of *Rhus chinensis* calculated by Bayesian inference (BI) of **(A)** cpDNA and **(B)** nuDNA. Numbers above the branches indicate the bootstrap values (>0.95). Clades of cpDNA and nuDNA representing haplotypes and genotypes are discussed in the text.

In the cpDNA phylogeny, eight haplotypes (M2–M4, M9–M11, and M19–M20) were clustered into clade I (Figure 2A). M21 and M8 were separately defined into clade II and clade III (Figure 2A). M1 independently belonged to clade IV, which was probably the ancestral haplotype (Figure 2A). Clade V included ten haplotypes (M5–M7, M14–M18, and M22–M23), while clade VI only contained two haplotypes (M12–M13) (Figure 2A). Based on nuDNA data, five clades were redefined (Figure 2B). Nine genotypes (N2, N4–N5, N7–N8, N11–N13, and N15) were clustered into clade I (Figure 2B). Clade II and III, each included two genotypes, namely, N6 and N14 existed in clade II, while N9 and N10 occurred in clade III (Figure 2B). N1 and N3 were individually defined into clade IV and clade V (Figure 2B). In addition, the divergence time of *R*. *chinensis* estimated with a strict molecular clock was highly consistent with that based on a relaxed molecular clock. According to the cpDNA phylogenetic tree, the crown age of *R. chinensis* was dated to be 4.2 ± 2.5 Mya, when the clade VI (P7 and P10–P11, Yunnan populations) split from all other clades (Figure 2A). Similarly, for the *LEAFY* gene, the crown age of *R. chinensis* was dated to be 4.8 ± 3.6 Mya (Figure 2B). Additionally, the clade III (P7 and P10–P11, Yunnan populations) diverged from clade I and clade II at 3.8 ± 3.0 Mya (Figure 2B).

### Ecological Niche Modeling

**FIGURE 3:**
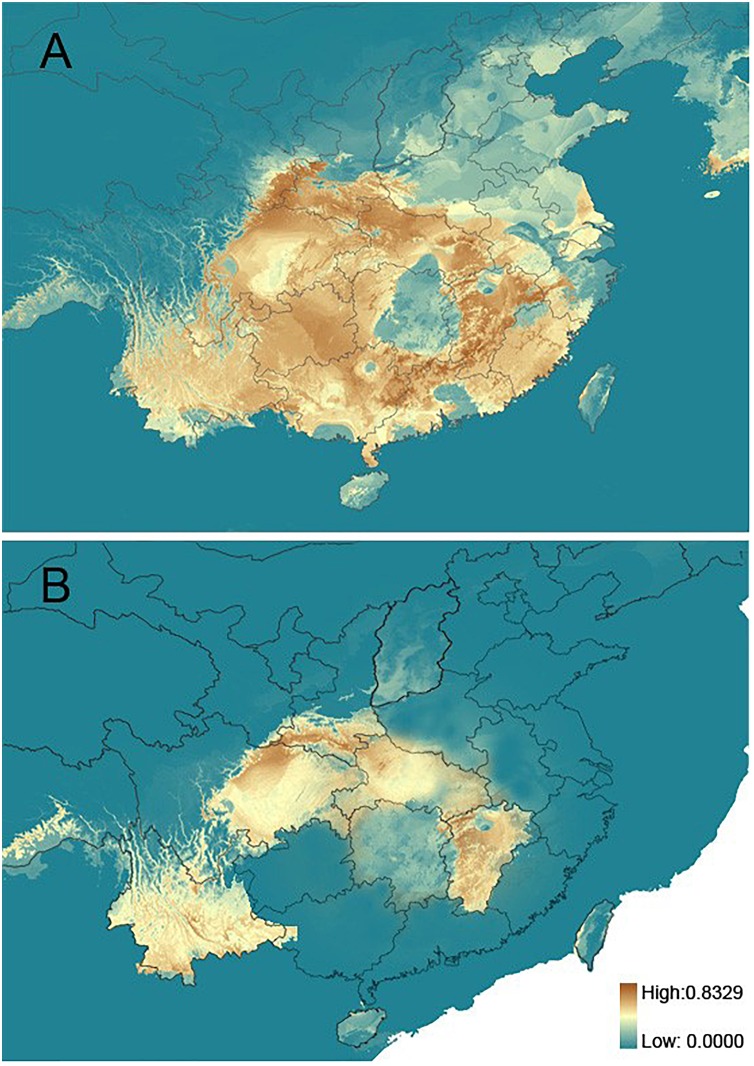
Maps showing the potential distribution by ENM. **(A)** The present. **(B)** Last glacial maximum.

The inferred past (LGM) and current distributions of *R. chinensis* are illustrated in (Figure 3). The AUC values based on both training and test presence data for the present and at the LGM were all higher than expected (not shown), which demonstrated good model performance. It was notable that the current distribution model indicated that *R. chinensis* mainly occurred in the warm temperate zone of China, which also suggested that it should occur in the same region during the LGM period (Figure 3A). In the comparison with the two simulated distributions, the LGM distribution model predicted that the species was mainly concentrated in Yunnan and central China including Shaanxi, Sichuan, Hubei, and Jiangxi provinces, and it had slightly shrunk in these regions during the LGM period (Figure 3B).

## Discussion

We did not detect a clear phylogeographic structure among the 19 populations of *R. chinensis* sampled in the present study. We found a somewhat lower differentiation among *R. chinensis* populations (*N*_ST_ = 0.382 for cpDNA, *N*_ST_ = 0.321 for nuDNA) compared to sympatric species such as *Platycarya strobilacea* (Chen S.C. et al., 2012) and *Cotinus coggygria* (Wang W. et al., 2014). Limited phylogeographic structure within a metapopulation may be due to high levels of geneflow and/or of geophysical connectedness (Avise et al., 1987). High levels of gene flow among *R. chinensis* populations may be due to the seed dispersal mechanism, which has been implicated in high levels of gene flow in many other plant species (e.g., Lopez et al., 2007; Song et al., 2013; Johnson et al., 2017). *R. chinensis* can produce 1000 seeds per plant on average, and the seeds are dispersed by animals, including mammals and birds, and by water (Huang and Qiu, 1994; Wang W. et al., 2014). Therefore, it is possible that relatively limited population differentiation may be due to the movement of seeds, including maternal and bi-parental genetic material, throughout the warm temperate zone. Geophysical connectedness within the range of *R. chinensis* may also be responsible for high levels of gene flow among populations. Stated another way, there may be limited barriers to dispersal. In the distributional area of *R. chinensis*, no obvious geographic barriers have been observed. Therefore, *R. chinensis* does not appear to be geographically isolated, allowing ecological niche modeling to be used in the assessment of species status (Li X.H. et al., 2012; Liu L. et al., 2014; Wang W. et al., 2014). Ecological niche models suggested the suitable habitats of *R. chinensis* were continuous in the present time while compressed during the LGM period, demonstrating multiple possible isolated glacial refugia (Figure 3). The response to impact of cold and warm times on the distribution of *R. chinensis* was validated in the simulation of ecological niche modeling, although we only used the simulated environment of current and LGM period (Figure 3). This pattern of range shifts indicated a likely scenario of repeated glacial compressions followed by interglacial expansions for *R. chinensis* during the Quaternary climatic oscillations. It is interesting that the geographic distribution of the cpDNA haplotypes differs from the nuDNA genotypes (Figure 2). Mismatch distributions between organellar DNA haplotypes and nuclear DNA genotypes have been reported in other groups such as *Sophora davidii* (Fan et al., 2013), *Cycas diannanensis* (Liu J. et al., 2015), and *Osteomeles schwerinae* (Wang Z.W. et al., 2015). Therefore, we thought that the forest birds and mammals were known as seed dispersers for many species in Anacardiaceae (Wang W. et al., 2014), which might have directly impacted the genetic structure with biparental inheritance.

The populations originated from Yunnan occurred at the China–Vietnam border and split from other clades at 4.2 ± 2.5 and 3.8 ± 3.0 Mya according to the cpDNA (clade VI) and nuDNA (clade III), respectively (Figure 2A,B). Early diverging populations in Yunnan have been detected in other genera or species such as *Ceratotropis* (3.62 Mya, Javadi et al., 2011), *Incarvillea sinensis* (4.4 Mya, Chen S. et al., 2012), and *Stuckenia filiformis* (3.93 Mya, Du and Wang, 2016). Within these species, the uplift of the QTP has been implicated as the main mechanisms of driving diversifications, but the estimated divergences were more recent than the last phase of the uplift (7–8 and 13–15 Mya; Harrison et al., 1992; Shi et al., 1998; Spicer et al., 2003). So, we thought that the geographical isolation of Yunnan populations was caused by the isolation of the QTP uplift in late Pliocene. Furthermore, the suitable climate in the temperate and subtropical zone could have subsequently facilitated the Pliocene-Pleistocene diversification of *R. chinensis* into different eco-geographic populations (Javadi et al., 2011).

Previous phylogeographic studies have widely supported hypotheses that climatic changes during the LGM forced plants into refugia within Central China, where they were protected by the QTP from the brunt of the ice age (Tian et al., 2009; Liu et al., 2012). After the glaciers retreated, the plants expanded their ranges rapidly (Hewitt, 2000; Li Z.H. et al., 2012; Qi et al., 2012). Our results showed that the range of *R. chinensis* had increased since the LGM (Figure 3) but did not support a rapid expansion based on the mismatch distribution (Figure 1) and Tajima’s *D* (1.19, 0.05 < *P* < 0.10 for cpDNA; 2.37, *P* < 0.01 for nuDNA). Refugia in the warm temperate China may have been dominated by evergreen forest or temperate deciduous forest during the LGM (Liu, 1988). Thus, southern Shaanxi, northern Sichuan, Yunnan, and Jiangxi could have supported *R. chinensis* during the LGM and been its main center of diversity. Just as *P. strobilacea* (Chen S.C. et al., 2012), *Cercidiphyllum* (Qi et al., 2012), and *C. coggygria* (Wang W. et al., 2014), the plants were slightly affected and were able to survive *in situ* at the period of the glaciation. So, the characterized phylogeographic structure of *R. chinensis* was consistent with the second hypothesis, which was that they survived *in situ* and occupied multiple localized glacial refugia during the glaciation.

## Conclusion

We used cpDNA and nuDNA sequences, and ecological niche modeling to investigate the evolutionary history of *R. chinensis* distributed in the warm temperate zone of China. The cpDNA and nuDNA data separately revealed six and five clades corresponding to the geographic regions. The divergence among haplotypes and genotypes of *R. chinensis* occurred at the Pliocene based on cpDNA and nuDNA data. Our ENMs showed enlarged potential distributions in the present compared to LGM, but we did not detect a sudden demographic expansion after the glaciation according to the molecular data. Our results suggest that *R. chinensis* was not affected by glacial cycles seriously and survived *in situ* and occupied a few main refugia.

## Author Contributions

ZR conceived and designed the research. YL and YZ collected the samples, performed the experiments, and conducted data analyses. XS and ZR drafted the manuscript. JW polished the manuscript. All authors read and approved the final manuscript.

## Conflict of Interest Statement

The authors declare that the research was conducted in the absence of any commercial or financial relationships that could be construed as a potential conflict of interest.

## Footnotes

1 http://www.cvh.org.cn

2 http://data.gbif.org

3 http://www.worldclim.org/
